# Feasibility of focused cardiac ultrasound training for non-cardiologists in a resource-limited setting using a handheld ultrasound machine

**DOI:** 10.5830/CVJA-2022-057

**Published:** 2022-12-14

**Authors:** Benjamin Acheampong, Joseph R Starnes, David Parra, Jonathan H Soslow, Benjamin Acheampong, Yaw A Awuku, Muktar H Aliyu

**Affiliations:** Department of Pediatrics, Division of Pediatric Cardiology, Vanderbilt University Medical Center, Nashville, Tennessee, USA; Department of Pediatrics, Division of Pediatric Cardiology, University of Nebraska Medical Center, Omaha, Nebraska, USA; Department of Medicine and Therapeutics, University of Cape Coast, Cape Coast, Ghana; Vanderbilt Institute for Global Health, Vanderbilt University Medical Center, Nashville, Tennessee, USA

**Keywords:** echocardiography, Ghana, training programmes, healthcare access

## Abstract

**Background:**

Heart disease remains a leading cause of morbidity and mortality, particularly in low- and middle-income countries. Access to diagnostic modalities is limited in these settings. Limited echocardiographic studies performed by non-cardiologists can increase access, improve diagnosis and allow for earlier medical therapy.

**Methods:**

Two internal medicine residents at a tertiary-level hospital in Ghana were trained to perform limited echocardiographic studies. Each trainee performed 50 echocardiograms and interpreted 20 studies across three predetermined timepoints. Interpretation was compared to expert interpretation.

**Results:**

Agreement improved over time. At the final evaluation, there was high agreement across all aspects: left ventricular structure (70%, kappa 0.52, p = 0.01), left ventricular function (80%, kappa 0.65, p = 0.004), right ventricular structure (90%, kappa 0.71, p = 0.002), right ventricular function (100%, kappa 1.00, p < 0.001), and presence of effusion (100%, kappa 1.00, p < 0.001).

**Conclusion:**

Non-cardiologists can be trained in focused echocardiography using handheld machines. Such training can increase access to diagnostic capabilities in resource-limited settings.

Both congenital and acquired heart disease remain important sources of childhood morbidity and mortality around the world. Congenital heart disease (CHD) accounted for more than 260 000 deaths in 2017, more than 180 000 of which were in infants less than one year of age.^1^

 Although high-income countries have seen improvement in CHD-related mortality of more than 50% since 1990, countries in the lowest quantile have improved by just 6%.1 Similarly, an estimated 319 400 deaths occurred due to rheumatic heart disease (RHD) in 2015.2 While RHD has essentially been eliminated in many high-income countries, rates remain as high as 10 per 1 000 in sub-Saharan Africa.^2^

 There is a significant difference in reported prevalence of CHD between high- and low-income countries (LIMCs), likely secondary to differences in access to healthcare and diagnostics.^3^ Although sub-Saharan Africa has the highest prevalence of RHD, prevalence is likely underestimated for the same reasons.^4^

Ischaemic heart disease and stroke are the leading causes of the cardiovascular disease burden, but RHD, cardiomyopathy and acute myocarditis remain important causes of morbidity and mortality.^5^ Rates of cardiac death are higher in LMICs, suggesting that later diagnosis and reduced access to care play a role in higher mortality rates.^6^,^7^ Furthermore, rates of cardiac disease in LMICs are difficult to quantify due to lack of data^5^ and are likely underestimated due to poor availability of diagnostic technologies.^8^

 Cardiovascular imaging in the developing world is limited by cost and expertise. Recent improvements in handheld ultrasound technology have the potential to reduce barriers to access and lead to better identification of paediatric and adult cardiac disease. Portable ultrasound, including for echocardiography, has shown promise in the triage, diagnosis and treatment of patients in LMICs.^9^ This technology has been used successfully by experts in LMICs in a variety of settings for adults^10^-^14^ and for RHD screening in children.^15^-^19^

As this technology has become more available, various efforts have aimed to train non-experts to perform focused handheld echocardiography. A number of studies have demonstrated acceptable accuracy of focused studies by non-echocardiographers in high-resource settings.^20^-^28^ In the LMIC setting, studies have included cardiologists,^11^,^14^-^17^ cardiology trainees,^10^,^13^ sonographers,^12^ and, more recently, nurses and other non-experts.^18^,^29^-^33^ These studies were performed only in adults,^11^-^14^ were performed by expert cardiologists,^15^-^17^ or were limited to the evaluation of RHD.^18^,^29^-^33^

 We hypothesised that non-experts, specifically internal medicine trainees, could use focused handheld echocardiography to successfully evaluate both children and adults. To our knowledge, this is the first study in an LMIC setting in which non-experts applied focused handheld echocardiography to such a broad patient group.

 Non-echocardiographers acquiring echocardiographic images need to be trained adequately in acquisition and interpretation to meet consistently high standards. The American Society of Echocardiography (ASE) has published guidelines on focused cardiac ultrasound, including training.^34^ The ASE recommends that this training include didactic education, hands-on experience and interpretation experience. The aim of this study was to assess the feasibility of a training programme for non-echocardiographers based on the ASE recommendations in resource-limited settings with handheld echocardiographic machines.

## Methods

Echocardiograms were performed on 100 patients, including children and adults, at the Cape Coast Teaching Hospital in Ghana between July and September 2018. The first 100 patients to consent were included, regardless of the reason for presentation to the hospital. Residents were selected for training based on their willingness to participate.

 The study was approved by the Cape Coast Teaching Hospital ethical review board. All patients who agreed to participate in the study were included. Participants gave appropriate consent and assent where applicable.

 The Lumify probe (Lumify, Philips, Amsterdam, the Netherlands) is a 1–4-MHz single-phased array cardiac transducer equipped with scanning software that is connected to an Android tablet interface. The transducer performs most of the beam forming, image acquisition and reconstruction processing. The smart device serves as the display screen and is connected to a cloud-based application. The touchscreen display allows users to tap to start functions, pinch and drag to zoom in and out, and swipe to expand the images. Images can also be wirelessly transferred to a picture-archiving and communication system.

 Two internal medicine residents at the Cape Coast Teaching Hospital in Cape Coast, Ghana were trained. The training consisted of a didactic component, which included instructional videos with a paediatric cardiologist available to answer questions, which was followed by proctored image acquisition, independent imaging, and interpretation of echocardiograms. Echocardiographic training focused on obtaining limited echocardiographic windows for qualitative assessment of left ventricular (LV) size and function, right ventricular (RV) size and function, and presence or absence of pericardial effusion. Colour flow, doppler and evaluation of valvular stenosis and regurgitation were also discussed, but were not the focus of the study and were therefore not included in the evaluation phase. This training model was based on ASE recommendations for focused cardiac imaging with handheld machines.^34^

 The didactic component consisted of training lectures, including basic ultrasound principles, basic cardiac anatomy and physiology, orientation to the machine, and demonstration of scanning, standard scanning windows and steps in interpreting echocardiographic studies (Table 1). During the didactic section, trainees were able to observe imaging by ultrasound technicians.

**Table 1:** Didactic content

Basic principles of ultrasound/echocardiography
Handling of portable echocardiogram machine
Cardiac anatomy and physiology
Imaging windows in echocardiography
Subjective assessment of LV structure and function
Subjective assessment of RV structure and function
Subjective assessment of atrial structure
Evaluation of pericardium

After the didactic and observation period, trainees began a 10-week practical training that included a minimum of 20 individual scans per trainee with interpretations (Table 2). Each scan included multiple grayscale images: two each from the parasternal long-axis, short-axis and apical four-chamber views over a preset two-second loop (Table 3). M-mode images from the parasternal short axis at the level of the papillary muscles and through the aortic root and left atrium were obtained over four beats.

**Table 2:** Elements of practical training

Two-dimensional imaging
Qualitative assessment of LV structure and function
Qualitative assessment of RV structure and function
Qualitative assessment of atrial structures
Presence and severity of pericardial effusion
M-mode
LV shortening fraction (parasternal short axis)

**Table 3:** Images obtained in protocol

*Imaging window*	*View*
Parasternal long axis	2D
Parasternal short axis	2D, M-mode
Apical 4-chamber	2D

 These training scans were performed under direct supervision by a third-year paediatric cardiology fellow. Immediate feedback was provided to help improve image quality and understanding for subsequent studies. After the first six-week proctored phase, each trainee performed additional training that included three weeks of proctored imaging and interpretations and a one-week period of independent imaging and interpretation.

Trainees reviewed images immediately after scanning and recorded interpretation in a secured REDCap database.^35^ Interpretation included qualitative analyses of LV and RV structure and function as well as evaluation of pericardial effusion. Each scan was independently interpreted by a paediatric cardiology fellow for comparison.

 Each trainee performed 50 echocardiograms and independently interpreted 20 randomly selected studies. Evaluations occurred at three predetermined timepoints. The first was following six weeks of proctored training (20 proctored studies performed and 10 interpreted studies per trainee), the second following an additional three weeks of proctored training (20 independent studies performed and five interpreted studies per trainee), and the last at the completion of the study (10 independent studies performed and five interpreted studies per trainee).

## Statistical analysis

All statistics were performed using Stata version 14.2 (StataCorp LP, College Station, TX). Kappa coefficients were calculated to measure inter-rater reliability. Predetermined cut-offs of < 0.00, 0.00–0.20, 0.21–0.40, 0.41–0.60, 0.61–0.80 and 0.81–1.00 indicating no agreement, poor, fair, moderate, substantial and almost perfect agreement were used. Interpretation of LV structure, LV function, RV structure, RV function, and pericardial effusion was compared to expert interpretation by a paediatric cardiology fellow.

## Results

Forty studies from patients aged seven to 75 years, of whom 72% were males, were interpreted and included in the analyses. Twentyeight echocardiograms (70%) had at least one abnormality in the five evaluated categories. LV abnormalities were more common than RV abnormalities (Table 4).

**Table 4:** Characteristics of echocardiograms read by paediatric cardiologist

*Variables*	*Normal*	*Mild dilation*	*At least moderate dilation*
LV structure, n (%)	18 (45)	15 (37.5)	7 (17.5)
RV structure, n (%)	36 (90)	1 (2.5)	3 (7.5)
	Normal	Mild depression	At least moderate depression
LV function, n (%)	21 (52.5)	5 (12.5)	14 (35)
RV function, n (%)	32 (80)	3 (7.5)	5 (12.5)
	Absent	Present	
Pericardial effusion, n (%)	33 (82.5)	7 (17.5)	

 Inter-rater reliability was generally moderate to strong and improved over successive evaluation periods, as measured by the kappa coefficient (Table 5). One notable exception was LV structure, for which kappa slightly decreased over successive timepoints, beginning at 0.67 during the first evaluation and ending at 0.52 during the final evaluation.

**Table 5:** Agreement in interpretation

	*Evaluation*	*1 (n=20)*		*Evaluation*	*2 (n=10)*		*Evaluation*	*3 (n = 10)*	
	*Agreement (%)*	*Kappa*	*p-value*	*Agreement (%)*	*Kappa*	*p-value*	*Agreement (%)*	*Kappa*	*p-value*
LV structure	80.0	0.67	< 0.001	80.0	0.64	0.002	70.0	0.52	0.01
LV function	60.0	0.23	0.07	80.0	0.55	0.03	80.0	0.65	0.004
RV structure	75.0	0.22	0.06	90.0	0.47	< 0.001	90.0	0.71	0.002
RV function	75.0	0.00	-	80.0	0.41	0.04	100.0	1.00	<0.001
Effusion	100.0	1.00	< 0.001	70.0	-0.15	0.70	100.0	1.00	< 0.001

 Similarly, percentage agreement was high throughout the study period and reached perfect agreement for some measurements. Although 70% agreement was found for pericardial effusion at the second timepoint, kappa was negative because 74% agreement was expected. Of note, only trivial and small pericardial effusions were missed.

 In order to continue supporting the residents, a gateway for image uploading was provided. This process allowed the cardiology fellow to continue to observe echocardiograms obtained in Ghana and to help the imagers with any questions or concerns, particularly images that were out of their scope of practice.

## Discussion

A short training course based on ASE recommendations was sufficient to adequately train non-cardiologist residents to perform focused limited echocardiography in a low-resource setting in Ghana over a 10-week period. There was generally high percentage agreement and inter-rater reliability compared to a paediatric cardiology fellow with echocardiographic experience. This study provides a potential model that could be expanded, both at the Cape Coast Teaching Hospital and in other similar settings.

 More than 90% of children with heart disease are born in parts of the world where adequate cardiac care is not available.^36^ The expansion of echocardiography beyond specialists can begin to bridge at least part of the gap across the continuum of cardiac care.

 This study adds to the growing literature that non-echocardiographers can demonstrate acceptable accuracy using focused cardiac ultrasound.^20^-^28^,^34^ Although studies in the global setting have traditionally utilised experienced cardiologists or sonographers, more recent studies have incorporated other providers, such as nurses and healthcare workers.^18^,^29^-^33^ However, these studies have been limited to the evaluation of RHD by increasingly limited protocols.

 To our knowledge, this is the first study, albeit in a very small group, to show that non-experts in LMIC settings can successfully perform and interpret general echocardiograms in both children and adults. This is an important expansion of studies conducted in high-income countries, as LMICs provide a specific set of logistics challenges, including new equipment, infrastructure and educational barriers.

 There were some notable exceptions to the generally high level of inter-rater reliability in the study. Most notably, kappa was 0.00 for RV function at the first timepoint and –0.15 for effusion at the second timepoint despite high percentage agreement. This is a known paradox for the kappa statistic when events, such as RV dysfunction or effusion, are rare in the data.^37^,^38^ The high percentage agreement shows that the trainees demonstrated sufficient accuracy despite the low kappa values in these isolated circumstances.

 As the application of handheld echocardiography evolves, the training of personnel not originally intended to perform focused cardiac studies is becoming increasingly important. Skills in acquiring and interpreting focused handheld cardiac images should be taught to novices via both didactic and proctored training prior to implementation in resource-limited settings. Expectations of trainees and subjects should be carefully managed, including the limitations of handheld echocardiogram.

The curriculum developed for this study was created to quickly train providers in basic echocardiographic assessment, but this curriculum can be duplicated in other underserved regions and modified to emphasise other areas of focus (for example, more focused assessment of valve disease for RHD and more focused assessment of wall-motion abnormalities for myocardial infarction). The didactic lectures have been recorded and can be distributed. The hands-on training is relatively short when compared with the potential improvement in access to care.

Our study is primarily limited by the small sample size. While the training model may be generalisable, only two residents at a single teaching hospital participated in the study, making comment on generalisability impossible.

 This study serves as a proof of concept that a short, multimodal training course can lead to skill acquisition in echocardiography. Future work will focus on adapting this model to larger groups, other geographic contexts and other types of acquired and congenital heart disease. Additionally, evaluation of trainees took place during and immediately following the training period. This timeline does not allow us to comment on the maintenance of skills over time. We plan to evaluate this schedule in future studies to determine the frequency of needed refresher trainings.

 We also report only qualitative assessment of the ventricular structure and function, as quantitative evaluation may not be available in resource-limited settings. In this context, abnormalities detected by non-cardiologists can be categorised into levels of severity with relative confidence, given the moderate to strong agreement in our study.

## Conclusion

Non-cardiologist residents can be trained in focused echocardiography in a low-resource setting using handheld echocardiographic machines. This capacity building could increase access to point-of-care focused cardiac ultrasound in resource-limited settings if successful in broader contexts.
